# Ursolic Acid Lactone Obtained from *Eucalyptus tereticornis* Increases Glucose Uptake and Reduces Inflammatory Activity and Intracellular Neutral Fat: An In Vitro Study

**DOI:** 10.3390/molecules26082282

**Published:** 2021-04-15

**Authors:** Norman Balcazar, Laura I. Betancur, Diana L. Muñoz, Frankly J. Cabrera, Adriana Castaño, Luis F. Echeverri, Sergio Acin

**Affiliations:** 1 GENMOL Group, Sede de Investigación Universitaria, University of Antioquia, Medellín 050010, Antioquia, Colombia; norman.balcazar@udea.edu.co (N.B.); lauraisabel.betancur@gmail.com (L.I.B.); frankly.cabrera@udea.edu.co (F.J.C.); 2 Department of Physiology and Biochemistry, Faculty of Medicine, University of Antioquia, Medellín 050010, Antioquia, Colombia; diana.munoz@udea.edu.co; 3 QOPN Group, Faculty of Exact and Natural Sciences, Sede de Investigación Universitaria, University of Antioquia, Medellín 050010, Antioquia, Colombia; adricast.09@gmail.com (A.C.); fernando.echeverri@udea.edu.co (L.F.E.)

**Keywords:** diabetes, ursolic acid lactone, macrophages, adipocytes, myocytes, hepatocytes

## Abstract

Obesity has a strong relationship to insulin resistance and diabetes mellitus, a chronic metabolic disease that alters many physiological functions. Naturally derived drugs have aroused great interest in treating obesity, and triterpenoids are natural compounds with multiple biological activities and antidiabetic mechanisms. Here, we evaluated the bioactivity of ursolic acid lactone (UAL), a lesser-known triterpenoid, obtained from *Eucalyptus tereticornis.* We used different cell lines to show for the first time that this molecule exhibits anti-inflammatory properties in a macrophage model, increases glucose uptake in insulin-resistant muscle cells, and reduces triglyceride content in hepatocytes and adipocytes. In 3T3-L1 adipocytes, UAL inhibited the expression of genes involved in adipogenesis and lipogenesis, enhanced the expression of genes involved in fat oxidation, and increased AMP-activated protein kinase phosphorylation. The range of biological activities demonstrated in vitro indicates that UAL is a promising molecule for fighting diabetes.

## 1. Introduction

Medicinal plants are a source of secondary metabolites that may be used to treat diseases. Ethnopharmacology and phytotherapy are valid strategies in treating obesity and type 2 diabetes mellitus (T2DM) [1]. Diabetes is a chronic disease that was calculated to affect 9.3% of adults aged 20–79 years (463 million people) in 2019 and this is estimated to increase to 578 million adults by 2030 and 700 million by 2045 [2]. Most people with diabetes have T2DM, which is largely the result of excess body weight and physical inactivity; moreover, obesity is associated with a ˃8-fold risk of T2DM [3]. Adipose tissue functions as an endocrine organ and regulates lipid storage and release. Hypertrophic and dysregulated adipose tissue produce increased amounts of proinflammatory adipokines and free fatty acids, leading to alteration of the mechanisms associated with insulin resistance and dyslipidemia, which affect adipose tissue itself and other tissues, including the muscles and liver [4]. An effective therapy for both the pathophysiological mechanisms and the complications of this disease is still being pursued, and natural products have become an important source of bioactive agents for T2MD treatment [5,6].

Triterpenoids are a family of molecules widely distributed in plants with multiple biological and pharmacological activities [7]. The triterpenoid content found in fruits and vegetables has been associated with different beneficial health effects [8]. These molecules present several biological activities, including anti-inflammatory, antioxidant, antiviral, antidiabetic, antitumor, hepatoprotective, and cardioprotective activities [8]. Eucalyptus is a plant used for treatment of diabetes mellitus in traditional medicine in some countries [9] and is a rich source of phytochemical constituents, including tannin and flavonoid, alkaloid, and propanoid compounds, which are found in the leaves, stems, and roots of the plant [10]. We have previously shown that leaf extracts from *Eucalyptus tereticornis* (Eu) have hypoglycemic/antidiabetic, hypolipidemic, and anti-inflammatory effects in cellular and animal models [11,12,13]. The Eu extract with the most beneficial properties contains three triterpenoids as the main molecules: ursolic acid (UA), oleanolic acid (OA), and ursolic acid lactone (UAL) [12,13]. UA has been described to have different anti-obesity/antidiabetic effects [14]. UA has anti-inflammatory activities and treatment with this molecule leads to reduction of liver weight and triglyceride content, decrease in plasma glucose levels, and improvement of adipocyte dysfunction [14,15]. OA also presents antioxidant and anti-inflammatory activities and exerts beneficial effects against diabetes by improving insulin response and inhibiting gluconeogenesis and promoting glucose utilization [16,17]. UAL is a less known triterpenoid that had previously been identified as a bioactive compound in Eu leaves [18], although the biological activities and pharmaceutical applications are unknown. We previously demonstrated that Eu extracts enriched in UAL have hypoglycemic effects in cellular models and modulate carbohydrate and lipid metabolism in a prediabetic mouse model, providing greater beneficial effects than purified Eu extracts with no UAL [11,13]. In this study, cell-culture models of macrophages, adipocytes, myocytes, and hepatocytes were used to investigate UAL activities, and we show for the first time that pure UAL has anti-inflammatory and antilipogenic effects and increases cellular glucose uptake.

## 2. Results

### 2.1. Effect of UAL on J774.A1 Macrophage Cell Line

**Figure 1:**
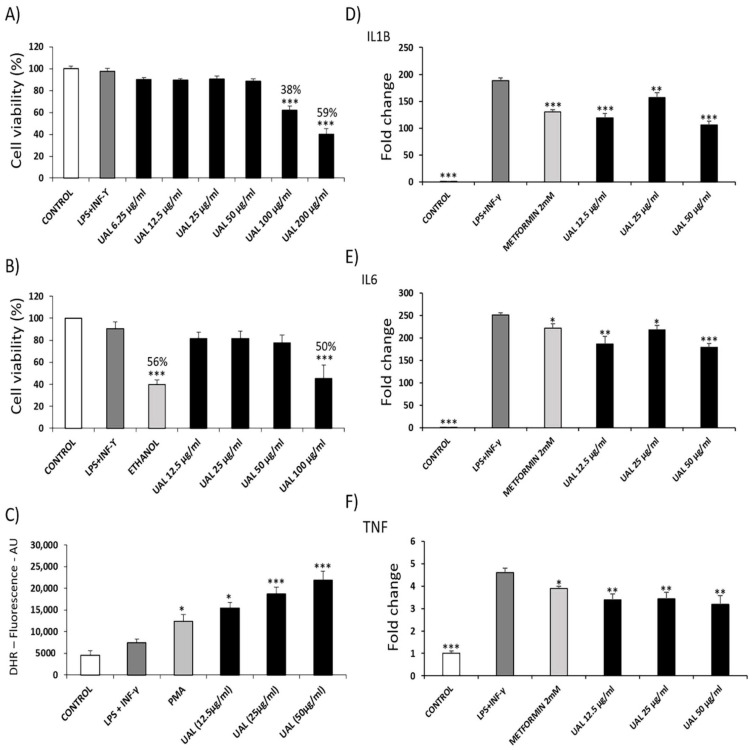
Effect of ursolic acid lactone (UAL) on cell viability, oxidative burst, and the expression of pro-inflammatory genes in activated J774A.1 macrophages. (**A**) J774A.1 cells were activated for 18 h and treated with various doses (0–200 μg/mL) of UAL. Cell viability was measured by the MTT assay after 6 h of treatment. The percentage of viable cells was calculated by defining the cell viability without treatment as 100%. Values are expressed as mean ± SEM of three independent experiments each one performed in triplicate. (**B**) J774A.1 cells were activated for 18 h and treated with various doses (0–100 μg/mL) of UAL. Cell viability was measured by 7-AAD fluorometric assay after 6 h of treatment. The percentage of viable cells was calculated by defining the cell viability without treatment as 100%. Values are expressed as mean ± SEM of three independent experiments each one performed in duplicate. (**C**) J774A.1 cells were activated for 18 h and treated with various doses (0–100 μg/mL) of UAL. Oxidative burst was quantified by flow cytometry using Dihydrorhodamine 123 (DHR) as qualitative marker of intracellular reactive oxygen species (ROS) after 6 h of treatment. PMA was used as positive control. Values are expressed as mean ± SEM of three independent experiments each one performed in duplicate. Relative expression of (**D**) Il1B, (**E**) Il6 and (**F**) Tnf mRNA transcripts is shown. J774A.1 cells were activated for 18 h and treated for 6 h with UAL. Metformin was used as positive control. Values are expressed as mean ± SEM of three independent experiments, normalized to the cyclophilin β gene expression. * *p* < 0.05, ** *p* < 0.01, *** *p* < 0.001, compared with LPS + INF-γ (activated cells) (ANOVA with Dunnett’s post hoc test).

The effect of UAL on cell viability was determined by MTT assay and flow cytometry-based analysis using 7-AAD dye (Figure 1A,B, respectively). Both techniques showed that 100 µg/mL UAL produced a significant reduction of 38% or more in viability of J774A.1 cells. For this cell line, UAL working concentrations up to 50 µg/mL were chosen. J774A.1 macrophages were activated with LPS and INF-γ for 24 h and were treated with UAL at 12.5, 25, and 50 µg/mL during the final 6 h of activation. Subsequently, they were then incubated with DHR 123, and the oxidative burst was quantified by flow cytometry. Treating activated macrophages with UAL caused a dose-dependent increase in reactive oxygen species (ROS) production (Figure 1C). To determine the action of UAL in the expression of proinflammatory genes we performed RT-PCR. J774A.1 cells treatment with UAL at 12.5, 25, and 50 µg/mL significantly decreased the transcriptional expression of for Il1B, Il6, and Tnf mRNA (Figure 1D–F).

### 2.2. Effect of UAL on C2C12 Myocyte Cell Line

**Figure 2:**
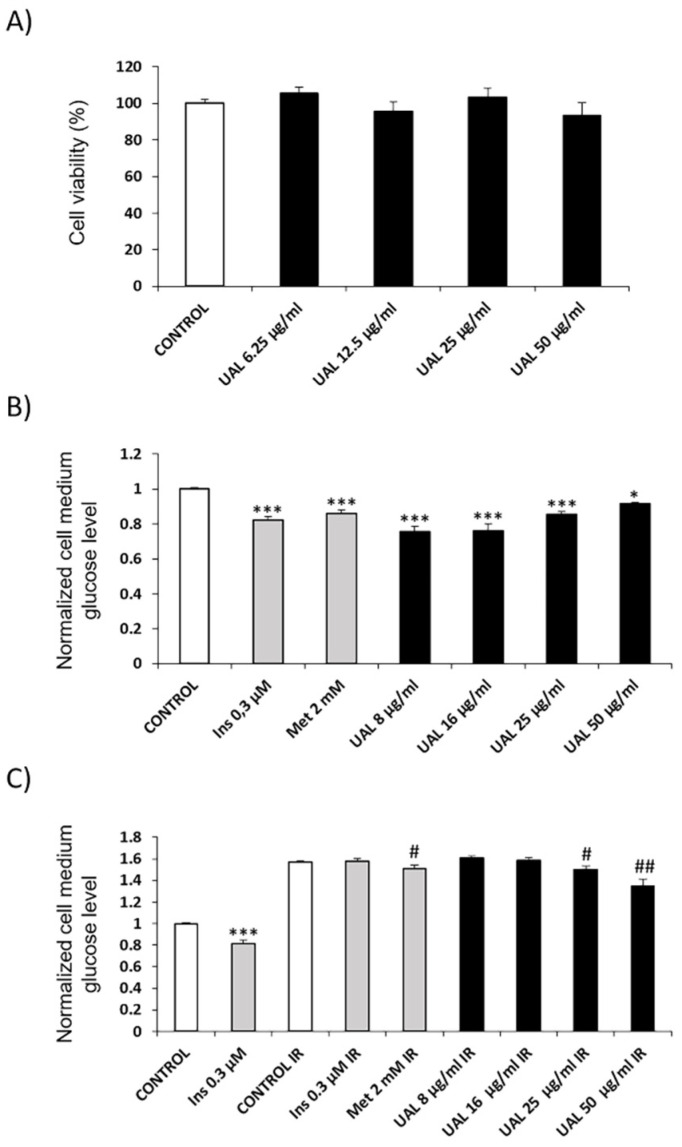
Effect of UAL on cell viability and glucose uptake in C2C12 cells. (**A**) C2C12 cells were treated with various doses (0–50 μg/mL) of UAL. Cell viability was measured by the MTT assay after 4 h of treatment. The percentage of viable cells was calculated by defining the cell viability without treatment as 100%. Values are expressed as mean ± SEM of three independent experiments each one performed in triplicate. (**B**) Non-insulin-resistant C2C12 cells were treated with various doses (8–50 μg/mL) of UAL. Insulin (Ins) and metformin (Met) were used as positive controls. Values are expressed as mean ± SEM of three independent experiments each one performed in triplicate. (**C**) Insulin-resistant C2C12 cells were treated with various doses (8–50 μg/mL) of UAL. Insulin and metformin were used as controls. Values are expressed as mean ± SEM of three independent experiments each one performed in triplicate. * *p* < 0.05, *** *p* < 0.001 compared with CONTROL; # *p* < 0.05, ## *p* < 0.01 compared with CONTROL IR (ANOVA with Dunnett’s post hoc test).

The effect of UAL on cell viability was determined by MTT assay. Cell viability was not significantly reduced at the concentrations used (Figure 2A). To evaluate the potential antidiabetic effects of UAL, the glucose uptake assay was performed in C2C12 myotubes. Figure 2B,C shows the effect of UAL on glucose uptake in non-insulin-resistant (Figure 2B) and insulin-resistant C2C12 cells (Figure 2C). In cells not previously exposed to palmitate, insulin and metformin increased glucose uptake as positive controls. UAL treatment also significantly increased medium glucose uptake; however, the lowest concentration used (8 µg/mL) was the most effective, with a 24% reduction in the supernatant glucose concentration compared with that of the control (Figure 2B). Exposure of C2C12 cells to palmitate induced insulin resistance. Figure 2C shows that when cells were previously exposed to palmitate, glucose concentration in response to insulin was not reduced compared with that in the control (Control R). Comparatively, when cells that had not been previously treated with palmitate were exposed to insulin (0.3 µM), there was a 19% reduction of glucose concentration in the supernatant compared with that in the control (Control). The effects of UAL on glucose uptake were also determined under conditions of insulin resistance. UAL increased glucose uptake in a dose-dependent manner. There was a greater increase of glucose uptake at 50 µg/mL and this was 14% higher in comparison with that of the control (Control R). Interestingly, this response was higher than the one obtained with a positive control (Metf 2 mM).

### 2.3. Effect of UAL on HepG2 Hepatocyte Cell Line

**Figure 3:**
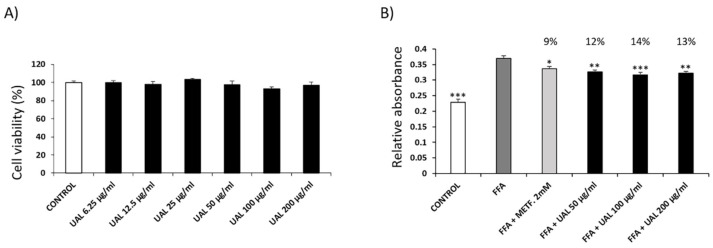
Effect of UAL on cell viability and lipid accumulation in HepG2 hepatocytes. (**A**) HepG2 cells were treated with various doses (0–200 μg/mL) of UAL. Cell viability was measured by the MTT assay after 72 h of treatment. Values are expressed as mean ± SEM of three independent experiments each one performed in triplicate. (**B**) HepG2 cells were loaded with FFAs (oleic acid:palmitic acid/2:1) and treated with UAL for 72 h. Metformin was used as positive control. HepG2 cells were stained with oil red and lipid accumulation was quantified. Values are expressed as mean ± SEM of four independent experiments each one performed in triplicate. * *p* < 0.05, ** *p* < 0.05, *** *p* < 0.001 compared with FFA group (ANOVA with Dunnett´s post hoc test).

The effect of UAL upon HepG2 cell viability was determined by MTT assay. HepG2 cell viability was not significantly reduced at concentrations used (Figure 3A). The effect of the UAL treatment on HepG2 lipid accumulation is shown in Figure 3B. Cells treated with this triterpene had significantly reduced lipid content at all concentrations used. A similar effect was observed following metformin treatment, which was used as positive control.

### 2.4. Effect of UAL on 3T3-L1 Adipocyte Cell Line

**Figure 4:**
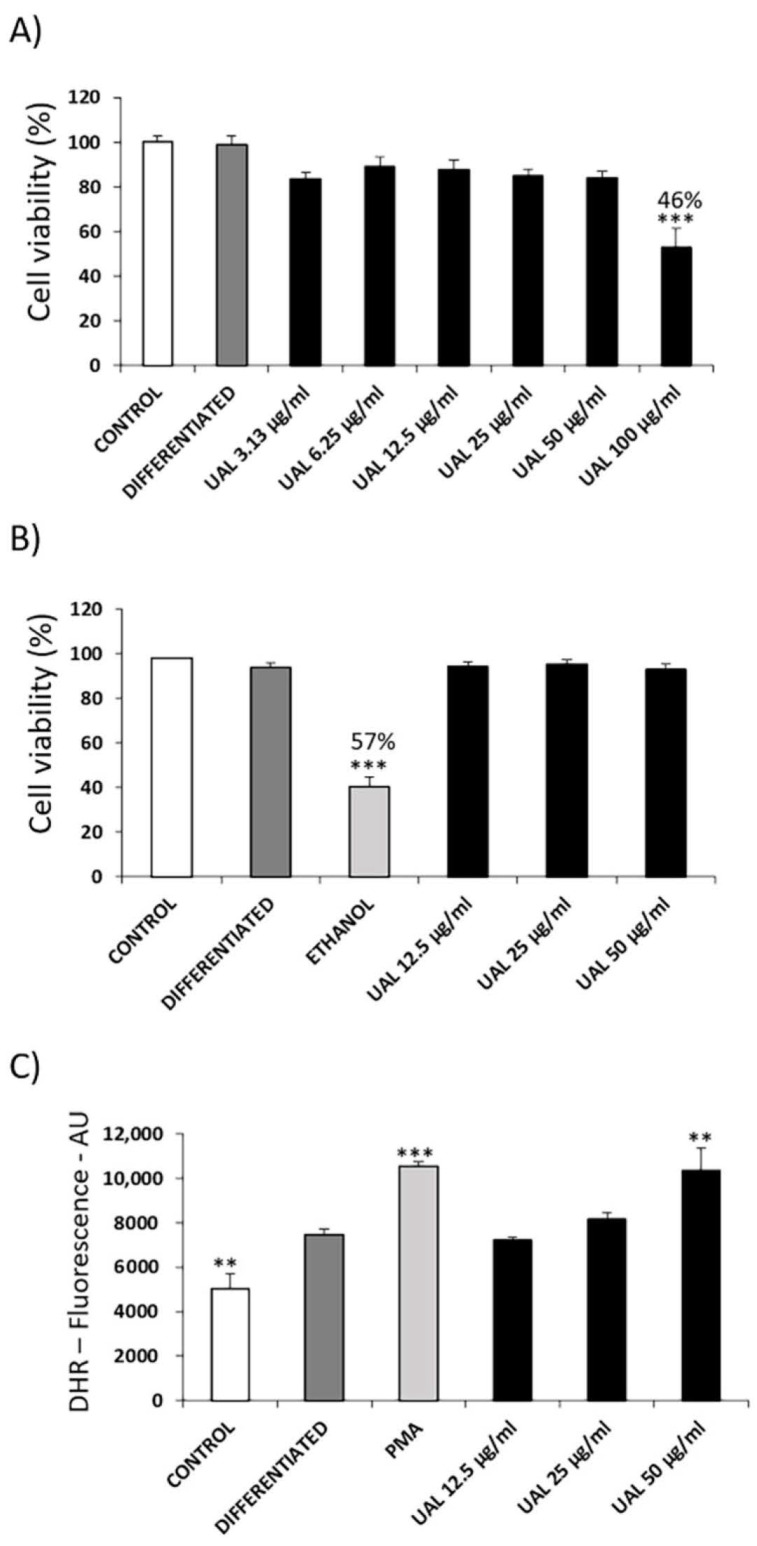
Effect of UAL on cell viability and oxidative burst in differentiated 3T3-L1 adipocytes. (**A**) 3T3-L1 cells were differentiated and treated with various doses (0–100 μg/mL) of UAL. Cell viability was measured by the MTT assay after 7 days of treatment. Values are expressed as mean ± SEM of three independent experiments each one performed in triplicate. (**B**) 3T3-L1 cells were differentiated and treated with various doses (0–50 μg/mL) of UAL. Cell viability was measured by 7-AAD fluorometric assay after 7 days of treatment. Values are expressed as mean ± SEM of three independent experiments each one performed in duplicate. (**C**) 3T3-L1 cells were differentiated and treated with various doses (0–50 μg/mL) of UAL. Oxidative burst was quantified by flow cytometry using Dihydrorhodamine 123 (DHR) as qualitative marker of intracellular reactive oxygen species (ROS) after 72 h of treatment. PMA was used as positive control. Values are expressed as mean ± SEM of three independent experiments each one performed in duplicate. ** *p* < 0.01, *** *p* < 0.001 compared with DIFFERENTIATED (ANOVA with Dunnett´s post hoc test).

**Figure 5:**
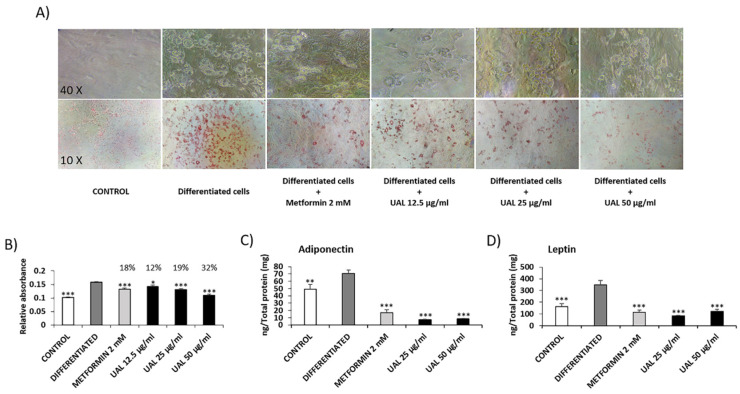
Effect of UAL on lipid accumulation in 3T3-L1 and protein expression in 3T3-L1 adipocytes. 3T3-L1 cells were differentiated and treated with UAL for 7 days. Metformin was used as positive control. (**A**) Photographs of 3T3-L1 adipocytes before and after Oil Red O staining. (**B**) 3T3L-1 cells were stained with oil red and lipid accumulation was quantified. Values are expressed as mean ± SEM of four independent experiments each one performed in triplicate. (**C**) Amount of adiponectin, and (**D**) amount of leptin present in the supernatant of the cells after 7 days of treatment. Values are expressed as mean ± SEM of three independent experiments each one performed in duplicate. * *p* < 0.05, ** *p* < 0.01, *** *p* < 0.001 vs. DIFFERENTIATED (ANOVA with Dunnett´s post hoc test).

The effect of UAL upon 3T3-L1 cell viability was determined by MTT assay and flow cytometry-based assay using 7-AAD dye (Figure 4). UAL at 100 µg/mL produced a significant reduction of 46% in viability of 3T3-L1 cells (Figure 4A). For this cell line, UAL working concentrations up to 50 µg/mL were chosen. Cytometry-based assay confirmed that UAL at 50 µg/mL was not cytotoxic in this cell line (Figure 4B). Oxidative burst was quantified by flow cytometry, and treatment of 3T3-L1 cells with UAL caused a significantly increased level of ROS at the highest concentration used (50 µg/mL) (Figure 4C). The effect of the UAL treatment on 3T3-L1 adipocyte lipid accumulation is shown in Figure 5. The lipid content of cells treated with UAL at 12.5, 25 and 50 μg/mL was significantly reduced by 12%, 19%, and 32%, respectively (Figure 5A,B). Metformin treatment, used as positive control, reduced fat load (18%). We assessed how treatment with this triterpenoid at the highest concentrations affected leptin and adiponectin protein expression in 3T3-L1 adipocytes. UAL significantly reduced expression of both adiponectin and leptin compared with that in differentiated adipocytes (Figure 5C,D).

**Figure 6:**
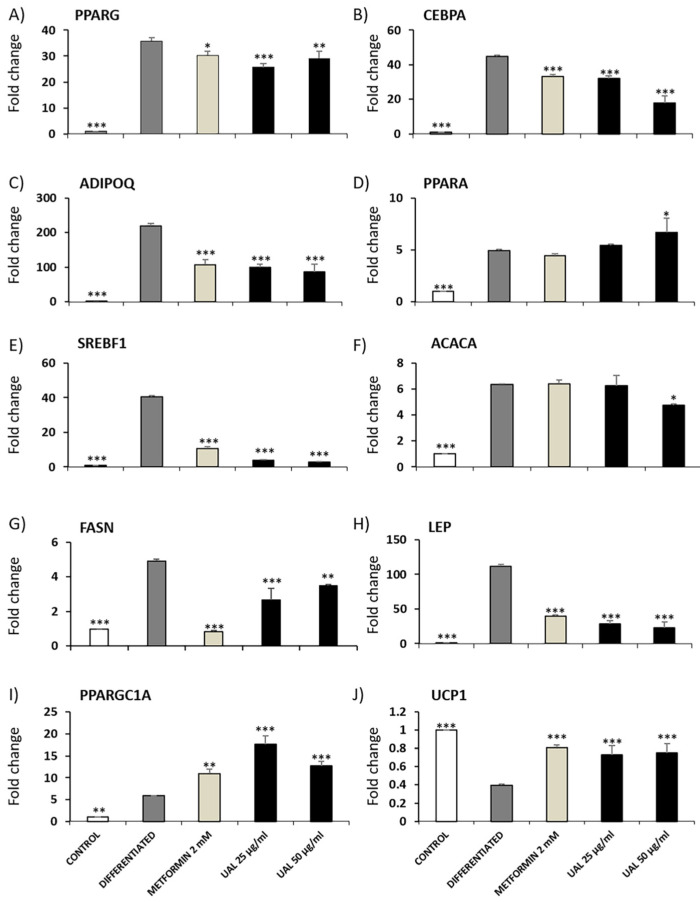
Effect of UAL on the expression of functional genes in 3T3-L1 cells. 3T3-L1 cells were differentiated for 4 days and treated with UAL for 24 h. Metformin was used as positive control. Relative expression of Pparg (**A**), Cebpa (**B**), Adipoq (**C**), Ppara (**D**), Srebf1 (**E**), Acaca (**F**), Fasn (**G**) Lep (**H**), Ppargc1a (**I**), Ucp1 (**J**) mRNA transcripts is shown. Values are expressed as mean ± SEM of three independent experiments. * *p* < 0.05, ** *p* < 0.01, *** *p* < 0.001 compared with DIFFERENTIATED (ANOVA with Dunnett´s post hoc test).

To verify whether UAL treatment altered adipocyte metabolism gene expression, we used qPCR to analyze the mRNA expression levels of Pparg, Cebpa, Adipoq, Ppara, Srebf1, Acaca, Fasn, Lep, Ppargc1a, and Ucp1 (Figure 6). The expression of Pparg, Cebpa, Adipoq, Srebf1, Fasn, and Lep mRNA was reduced with UAL treatment at 25 and 50 µg/mL compared with that of the untreated differentiated group (Figure 6A–C,E,G,H). Metformin, used as positive control, also downregulated expression of the genes encoding these proteins. Expression of Acaca mRNA was only reduced with treatment by UAL at the highest concentration (Figure 6F). UAL and metformin treatments upregulated expression of Ppargc1a and Ucp1 mRNA levels (Figure 6I,J); however, only UAL at 50 µg/mL significantly increased the expression of Ppara mRNA compared with that of the untreated differentiated group (Figure 6D).

**Figure 7:**
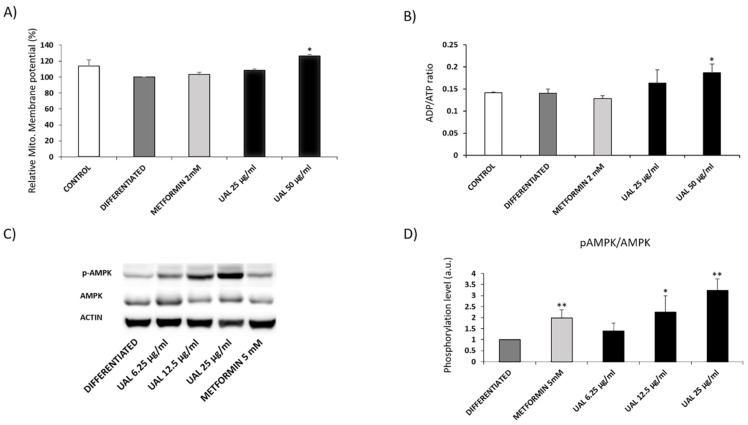
Effect of UAL on differentiated 3T3-L1 mitochondria and cellular AMPK expression. 3T3-L1 cells were differentiated and treated with UAL for 72 h. (**A**) Mitochondrial membrane potential was determined by quantification of Mito tracker-loaded cells fluorescence. Values are expressed as mean ± SEM of three independent experiments each one performed in duplicate. (**B**) ADP/ATP ratio in 3T3-L1 adipocytes treated with UAL. Values are expressed as mean ± SEM of three independent experiments each one performed in triplicate (**C**) Representative western blots of phosphorylated AMPK in differentiated 3T3-L1 cultures treated with UAL. (**D**) Quantitative analysis of the immunoblots of p-AMPK normalized with total AMPK. Values are expressed as mean ± SEM of three independent experiments. * *p* < 0.05, ** *p* < 0.01 compared with DIFFERENTIATED (ANOVA with Dunnett´s post hoc test).

Figure 7A shows the effect of UAL on mitochondria membrane potential (ΔΨm) in 3T3-L1 adipocytes treated with the triterpene for 72 h. The results showed a significant increase of ΔΨm in cells treated at 50 µg/mL compared with that of the differentiated group. Treatment with UAL at 50 µg/mL significantly increased the cellular ADP/ATP ratio (Figure 7B). Metformin treatment did not modify either of the two parameters. To further elucidate the molecular mechanism of the effect of UAL, we examined the activation of AMP-activated protein kinase (AMPK). UAL treatment enhanced the phosphorylation of AMPK in 3T3-L1 cells in a dose-dependent manner (Figure 7C,D). Similarly, metformin increased AMPK phosphorylation compared with that in differentiated cells.

## 3. Discussion

**Figure 8:**
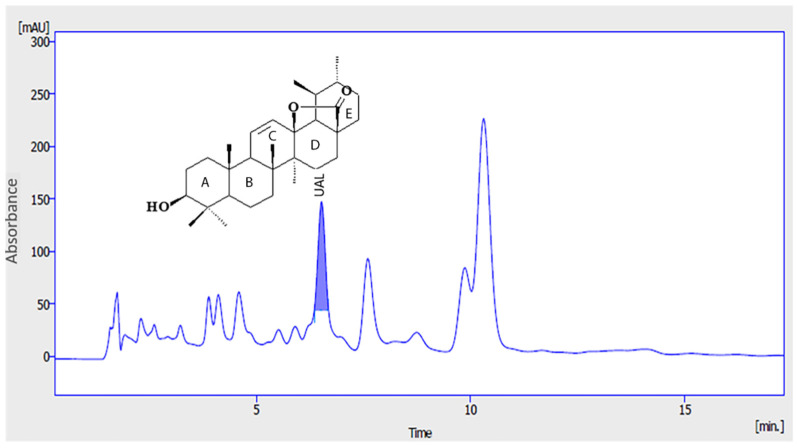
Chromatogram of Eu extract. Chromatographic profile of *E. tereticornis* leaves extract. UAL: Ursolic acid lactone.

Expansion of adipose tissue (AT) in obesity leads to severe adipocyte metabolic alterations that result in chronic inflammation. Proinflammatory M1 macrophages can constitute up to 40% of all AT cells that secrete a range of proinflammatory cytokines and impair suppression of free fatty acid release in response to insulin. Both of these are key factors for the development of insulin resistance in AT and other tissues such as the muscles and liver [19,20]. In this work, we analyzed the effect of UAL in macrophage, myocyte, hepatocyte, and adipocyte cellular models of the tissues involved in the development of insulin resistance and T2DM, focusing on adipocytes since AT is the primary whole-body regulator of lipid and glucose homeostasis. Pentacyclic triterpenoids are compounds with antioxidant, anti-inflammatory and antidiabetic activities [7,21]. Here, for the first time we showed that bioactivity of UAL a lesser known member of this family of natural molecules. UAL is a derivative of UA, which possesses important biological effects, although its clinical application is limited by its bioavailability and solubility [22]. We analyzed UAL cytotoxicity in different cell lines and we found that there was no toxic effect with treatment with UAL up to 50 µg/mL in mouse adipocyte, myocyte and macrophage cell lines and up to 200 µg/mL in a human hepatocyte cell line (Figure 1, Figure 2, Figure 3 and Figure 4). Interestingly, modification in ring D or E of UA, as occurs in UAL (Figure 8), results in a decline of cytotoxicity [23]. Our results confirm that UAL showed less cytotoxicity than UA, at least in the 3T3-L1 cell line, where it has been described that 20 µM (≈9 µg/mL) UA treatment produced a 50% reduction in cell viability [24], and the J774.A1 cell line, where 50 µg/mL UA treatment produced more than 20% reduction in cell viability (data not shown). Here, we showed that UAL treatment reduced the expression of genes encoding three proinflammatory proteins (Figure 1). These results are consistent with the anti-inflammatory activities of triterpenoids described [21]. Other pentacyclic triterpenoids such as UA or lupeol show a similar effect in LPS-activated macrophages and decreased the production of proinflammatory cytokines, including TNF-α, IL-6, and IL-1β [25,26]. A molecular target of most of triterpenoids, including UA and lupeol, is nuclear factor-κB (NF-κB), a transcription factor that is essential for inflammatory and innate immune responses [27]. The response observed after treatment with UAL suggests that this triterpenoid may have NF-kB as one of its targets.

UAL treatment caused a dose-dependent increase in ROS levels in macrophages and adipocytes (Figure 1 and Figure 4). Other studies have shown that lower doses of triterpenoids such as UA or OA (up to 10 µg/mL) enhanced ROS production in macrophages [28,29]. As previously reported, mitochondria produce significant amounts of ROS and are a primary target of ROS damage. High levels of ROS and poor antioxidant response are often associated with oxidative stress and lipid, protein and DNA damage. Excess levels of ROS also lead to the mitochondrial permeability transition pore complex to open causing depolarization of mitochondrial membrane potential [30,31,32]. Our results showed that UAL treatment, despite significantly increasing ROS production in macrophages and adipocytes at 50 µg/mL, did not affected the integrity of the cell membranes, as confirmed by 7-AAD-dye flow cytometry of both cell lines (Figure 1 and Figure 4). UAL also did not cause a depolarization of the adipocyte mitochondrial membrane, and on the contrary, increased the mitochondrial membrane potential (Figure 7). ROS production not only produces cell damage (i.e., oxidative stress) but also is involved in regulating signaling pathways that affect normal physiological and biological responses. Increased ROS production following UAL treatment may play this secondary role in adipocytes and macrophages, taking into account that expression of Il1B, Il6, and Tnf mRNA in macrophages was reduced by treatment with UAL, and ROS is required for the release of proinflammatory cytokines to effect an appropriate immune response [32,33].

Anti-inflammatory strategies have been shown to be effective in improving obesity-induced insulin resistance in murine models, however the effects observed are modest [19]. It is important to find therapies that can target different cells. UAL also has potential hypoglycemic effects, and we have shown that UAL treatment increased glucose uptake in insulin non-resistant myotubes as well as in a cellular model of insulin resistance in comparison with metformin used as positive control (Figure 2). These results confirm our previous work when we showed that an extract from *E. tereticornis* rich in UAL increased glucose uptake in C2C12 cells [11] and are consistent with other studies where triterpenoids such as UA or corosolic acid increase glucose uptake in myotubes [34,35].

Adipocytes maintain energy homeostasis, and the liver plays an important role in the regulation of blood sugar. An excessive increase in adipocyte size or numbers becomes a risk factor for the development of T2DM and fatty liver disease [36]. UAL treatment reduced lipid accumulation in HepG2 and 3T3-L1 cells (Figure 3 and Figure 5). Pentacyclic triterpenoids have been shown to attenuate adipogenesis and stimulate fatty acid oxidation [37]. UA and OA reduction of lipid accumulation in 3T3-L1 cells and the UA effect can be partially explained by the activation of 5′ AMPK. In our study, we explored in more detail how UAL affected adipocyte metabolism. As we have discussed, UAL treatment increased the mitochondrial membrane potential (ΔΨm). ΔΨm generated by proton pumps is an essential component in the process of making ATP. However, the increase in ΔΨm is accompanied with an increase of the ADP/ATP ratio after UAL treatment (Figure 7). Long-lasting changes in normal levels of ΔΨm may induce loss of cell viability, and after we confirmed this is not the case, the fluctuation in the levels of ΔΨm and ATP in 3T3-L1 cells may be interpreted as part of the normal physiological activity [38]. Interestingly, the reduction of ATP may explain the activation of AMPK after UAL treatment. AMPK is a major regulator of cellular energy homeostasis and sustained AMPK activation reprograms cells to limit glucose and lipid synthesis and favor oxidation of fatty acids as an energy source. AMPK is activated in response to cellular energy status by sensing increases in the AMP:ATP and ADP:ATP ratios and restores energy balance by inhibiting anabolic processes and promoting catabolic processes [39]. UAL increased the phosphorylation of Thr172 in a dose-dependent manner (Figure 7). This phosphorylation is required for full activation of AMPK [39]. AMPK is also a molecular target activated by other triterpenoids, such as UA, for reduction of lipid accumulation in 3T3-L1 cells [24] and inhibits transcription factors that activate glycolytic and lipogenic cellular programs. One of the most important of these is sterol regulatory element-binding protein 1 (SREBP1), a master transcriptional regulator of lipid synthesis [39]. UAL treatment suppressed Srebf1 gene expression and then downregulated Pparg and Cebpa gene expression (Figure 6). These three genes code for master regulators of adipocyte differentiation and promotion of the biosynthesis of fatty acids in 3T3-L1 cells [40,41]. Furthermore, UAL treatment suppressed adiponectin production (Figure 5 and Figure 6). These results are consistent with those from 3T3-L1 fibroblasts where overexpressed adiponectin increased the expression of Pparg, Cebpa, and Srebf1 mRNA and adipocyte differentiate occurred more rapidly with a higher accumulation of lipid drops [42]. Reduced intracellular fat accumulation by treatment with UAL may be partially explained by modification of the expression of the genes (Figure 5 and Figure 6) associated with downregulated expression of Fasn and Acaca mRNA (Figure 6), which encodes proteins involved in fat synthesis. UAL treatment also upregulated the expression of mRNA of Ppargc1a, Ppara, and Ucp1, which are highly expressed in brown fat and associated with fatty acid oxidation [43]. PPARGC1A is a protein associated with the brown fat cell phenotype, and PPARGCIA expression levels during adipocyte differentiation are progressively suppressed; this effect is enhanced by adiponectin overexpression. UAL treatment not only prevented the inhibition of Ppargc1a mRNA expression but significantly increased this, with the upregulation of the expression of Ucp1 mRNA, which encodes a protein associated with the brown fat cell phenotype (Figure 6) [43]. UAL treatment also increased the expression of Ppara, which encodes a protein that improves insulin resistance, ameliorates obesity, and cooperates with PPARGC1A to control lipid oxidation and thermogenesis in the brown adipose tissue [44]. Finally, in obesity conditions a large amount of leptin is secreted by adipocytes and this is associated with the inflammation state [45]. In this regard, UAL treatment has beneficial effects reducing leptin expression at both the gene and protein levels (Figure 5 and Figure 6).

## 4. Materials and Methods

### 4.1. Ursolic Acid Lactone (3β-hydroxy-urs-11-en-28,13β-olide) Purification

UAL is not available commercially and it was obtained from Eu leaves, after extraction with organic solvents (ethanol and methanol), fractionation in Sephadex LH-20, and repeated thin layer chromatography, using 0.25 mm silica gel plates and a mixture of hexane-ethyl acetate (1:1, *v*/*v*), as previously reported [11]. The compound was identified by 1H NMR and 13C NMR, in an AMX300 spectrometer (Bruker BioSpin GmbH, Rheinstetten, Germany) operating at 300 MHz and was used like a secondary standard in the following UAL purifications [12]

The presence in the extracts was detected through co-injection in the HPLC to determine the Rt and then compared with the chromatogram of the extract (Figure 1). Similarly, UAL was quantified by means of HPLC on a VWR Hitachi Chromaster 600 chromatograph set coupled with a diode array detector (DAD) using a XDB C18 column (4.6 × 150 mm i.d., 5 µm particle size) (Phenomenex, Torrance, CA, USA). A mixture of methanol: water: acetic acid 88:12:0.05 (*v*/*v*/*v*) was used as a mobile phase. The eluent flow rate was 1.0 mL/min. The column temperature was 25 °C. The data were collected in a wavelengths of 206, 254 and 300 nm. The quantitative analysis was conducted using the Clarity software v.7.4 (DataApex, Prague, Czech Republic). For extracting UAL from Eu dried leaves, the use of ethanol and ethyl acetate as solvents performed better (0.027%) than the use of methanol (0.015%).

All experimental analyses were conducted in accordance with the guidelines of the United States Pharmacopeia (USP 32), the 27th edition of the National Form (NF 27) (United States Pharmacopeial, 2009) and the International Conference on Harmonization (ICH) (ICH, 2005). The analytical method for obtaining UAL was validated for linearity, precision, accuracy, range and selectivity, which are the parameters used for the quantification of the main components. UAL used in this study were prepared in accordance with the guidelines of the European Medicines Agency (EMA). UAL was reconstituted in dimethyl sulfoxide (DMSO) at 25 mg/mL (stock solution).

### 4.2. Cell Cultures

J774A.1 (TIB-67™) mouse macrophage cells, C2C12 (ATCCCRL-1772) mouse muscle cells, 3T3-L1 (CL-173™) mouse fibroblast cells, and HepG2 (ATCC^®^ HB-8065™) human hepatic cells were purchased from ATCC (Manassas, VA, USA). Cells were cultured in Dulbecco’s Modified Eagle’s Medium (DMEM) with 10% fetal bovine serum (FBS), 2 mM glutamine and 1% penicillin/streptomycin (Sigma- Aldrich, St. Louis, MO, USA) at 37 °C and 5% CO_2_. HepG2 cells were cultured with 5.5 mM glucose (Sigma- Aldrich, St. Louis, MO, USA) (Growth medium 1—GM1); J774.A1, 3T3-L1, and C2C12 cells were cultured with 25 mM glucose (Growth Medium 2—GM2).

### 4.3. Macrophage Cell Culture and Activation

J774A.1 cells were cultured in GM2. At 80% confluence J774A.1 cells were incubated in GM2 containing 100 ng/mL lipopolysaccharide (LPS) and 20 ng/mL interferon gamma (IFN-γ) (activation medium—AM) (Sigma- Aldrich, St. Louis, MO, USA) for 24 h. Treatments were added during the last 6 h. MTT cell proliferation, 7-Amino Actinomycin D (7-AAD) cell viability, and Dihydrorhodamine (DHR) flow cytometry assays were performed. RNA was collected to evaluate inflammation related gene transcription.

### 4.4. Adipocyte Cell Culture and Differentiation

3T3-L1 pre-adipocytes were cultured in GM2. Differentiation was induced 2 days post-confluence by adding GM2 containing 0.5 mM 3-isobutyl-1-methylxanthine (IBMX), 0.25 μM dexamethasone, 2 μM Rosiglitazone (Sigma- Aldrich, St. Louis, MO, USA) and 1 μg/mL insulin (Novo Nordisk, Bagsværd Denmark). After 2 days of incubation, medium was replaced with GM2 containing 1 μg/mL insulin. Two days later, medium was replaced by GM2 and incubated for another 7 days with the different treatments (Replacing it every 2 days). MTT cell proliferation, 7-Amino Actinomycin D (7-AAD) cell viability, Dihydrorhodamine (DHR) and Mito Tracker flow cytometry assays were performed. Cells were stained to evaluate intracellular triglyceride (TG) accumulation, supernatants were collected to evaluate adipokine concentration, RNA was purified to evaluate functional gene transcription and protein was extracted for western blot analysis.

### 4.5. Myotube Cell Culture and Differentiation

C2C12 cells were cultured in GM2 and were differentiated into myotubes using DMEM with 5.5 mM glucose, 2% Horse Serum, 2 mM glutamine, and 1% penicillin/streptomycin (Growth medium 3—GM3) for 4 days. For non-insulin-resistant cells, medium was replaced by GM3 and incubated for another 4 h with the different treatments. To develop a model of insulin-resistant cells, 4-day differentiated myotubes cells were preincubated for 2 h in DMEM with 5.5 mM glucose without FBS and supplemented with 1% BSA and then incubated for 18 h in DMEM with 5.5 mM glucose without FBS, 1% BSA, 0.75 mM palmitate, and 200 mM insulin. C2C12 myotubes were incubated for 4 h in 500 µL DMEM, 5.5 mM glucose, and UAL. MTT cell proliferation assay was performed and supernatants were collected to evaluate glucose concentration.

### 4.6. Hepatocyte Cell Culture and Steatosis Induction

HepG2 cells were cultured in GM1. Fatty acid (FA) was prepared as FA-bovine serum albumin (BSA) complex with the ratio of oleic acid (OA): palmitic acid (PA) = 2:1 (Sigma- Aldrich, St. Louis, MO, USA). OA and PA were dissolved in 1% BSA in low glucose DMEM by stirring 2 h at 370 C. In vitro steatosis was induced by 0.5 mM of FA. HepG2 cells were treated with FA and UAL for 72 h. MTT cell proliferation assay was performed and cells were stained to evaluate intracellular TG accumulation.

### 4.7. Cell Viability

Adipocytes, myotubes, macrophages and hepatocytes were treated at different concentrations of UAL according to the cell type (3.13, 6.25, 12.5, 25, 50, 100 and 200 µg/mL) and a MTT Cell Viability Assay Kit (Sigma-Aldrich, St. Louis, Mo, USA) was used. MTT was added to the cells for 2 h. After that time, formazan crystals were dissolved by adding DMSO. Absorbance was measured at 570 nm in a Varioskan ™ LUX microplate multilector (Thermo Fisher Scientific, Waltham, MA, USA). Cell viability of adipocytes and macrophages were confirmed using 7-AAD Flow cytometry. Cells were harvested, wash with PBS twice and resuspended in PBS with 4% FBS and 0.2 µg/mL 7-AAD, incubated for 15 min at 37 °C and analyzed using BD LSRFortessa™ (BD) Cytometer (excitation 488 nm, emission 647 nm)(BD, Franklin Lakes, NJ, USA).

### 4.8. Determination of Oxidative Stress in J774.A1 and 3T3-L1 Cells

Dihydrorhodamine 123 (DHR) was applied as qualitative marker of intracellular reactive oxygen species (ROS). J774A.1 macrophage cells were activated and treated for 6 h, and 3T3-L1 adipocytes were differentiated and treated for 72 h. Phorbol 12-myristate 13-acetate (PMA) was used as positive control. Cells were re-suspended in PBS with 0.001 mM DHR, incubated for 15 min and analyzed using BD LSRFortessa™ (BD) Cytometer (excitation 488 nm, emission 530 nm).

### 4.9. RNA Extraction and Real-Time PCR in J774.A1 and 3T3-L1 Cells

Total RNA was extracted from cells with the RNeasy kit coupled with DNase treatment for genomic DNA removal (QIAGEN, Valencia, CA, USA), and reverse transcription reaction was performed with 500 ng total RNA, 50 ng/μL random hexamers, 10 mM dNTP Mix, 20 mM Tris-HCl pH 8.4, 50 nM KCl, 2.5 mM MgCl2, 40 U/μL RNaseOut, and 200 U/μL SuperScript III RT (Invitrogen, Waltham, MA, USA), according to the manufacturers’ instructions. Real-time quantitative PCR (qPCR) analyses were performed with 50 ng cDNA and 600 nM sense and antisense primers (Integrated DNA Technologies, Coralville, IA, USA) in a final reaction volume of 25 μL using the Maxima SYBR Green/ROX qPCR Master Mix (Thermo-Fisher Scientific, Waltham, MA, USA) and the CFX96 real-time PCR detection system (Bio-Rad, Hercules, CA, USA). The program for thermal cycling was 10 min at 95 °C, followed by 40 cycles of 15 s at 95 °C, 30 s at 53 °C, 58 °C, 59 °C, 60 °C, 64 °C or 68 °C, and 30 s at 72 °C. Results were normalized to the cyclophilin B (Ppib) expression level. For macrophages, the expression of the inflammatory marker genes Tumor Necrosis Factor (Tnf), Interleukin 1 Beta (Il1B) and Interleukin 6 (Il6) was evaluated, and for adipocytes, the expression of Peroxisome Proliferator Activated Receptor Gamma (Pparg), CCAAT Enhancer Binding Protein Alpha (Cebpa), Adiponectin (Adipoq), Peroxisome Proliferator Activated Receptor Alpha (Ppara), Sterol Regulatory Element Binding Transcription Factor 1 (Srebf1), Acetyl-CoA Carboxylase Alpha (Acaca), Fatty Acid Synthase (Fasn), Leptin (Lep), PPARG Coactivator 1 Alpha (Ppargc1a), Uncoupling Protein 1 (Ucp1) was evaluated and the relative amount of the whole mRNA was calculated using the comparative or ΔΔCt method. All the sequence-specific oligonucleotide primers (Supplementary Material—Table S1) were obtained from Invitrogen.

### 4.10. Measurement of Glucose Concentration in C2C12 Cells

C2C12 myotubes were incubated for 4 h in GM2 with treatments. Then, 500 µL of supernatant were collected, and glucose concentration was measured using the Glucose Oxidase Assay Kit (Invitrogen, Waltham, MA, USA). To calculate glucose utilization, the remaining glucose in the culture medium after incubation with controls and UAL was subtracted from the initial amount of glucose (5.5 mM).

### 4.11. Measurement of Triacylglycerol Concentration in 3T3-L1 and HepG2 Cells

3T3-L1 cells were differentiated and treated for 7 days and HepG2 were fat-loaded and treated for 72 h. Then, cells were washed with PBS and fixed in 10% formaldehyde at room temperature for 1 h. Cells were washed with 60% isopropanol and completely dried. The fixed cells were stained with Oil Red-O solution (Sigma-Aldrich, St. Louis, MO, USA) at room temperature for 30 min and washed with water. 3T3-L1 cells were photographed with a 10× and 40× magnification. The stain of lipid droplets was extracted with 100% isopropanol and the absorbance was measured at 492 nm in a Varioskan™ LUX multimode microplate reader (Thermo-Fisher Scientific, Waltham, MA, USA).

### 4.12. Quantification of Adipokines in 3T3-L1 Cells

Leptin (ab100718) and Adiponectin (ab108785) kits (Abcam, Cambridge, UK) were used for the quantitative measurement of mouse adipokines in culture medium after cells treatment. The absorbance of each sample was measured using a Varioskan™ LUX multimode microplate reader (Thermo-Fisher Scientific, Waltham, MA, USA). Results were normalized to the total protein concentration. Assays were performed according to the manufacturers´ instructions.

### 4.13. Mitochondrial Membrane Potential in 3T3-L1

3T3-L1 preadipocytes were differentiated and treated for 72 h, then cells were re-suspended in PBS and incubated with 20 mM of Mitotracker, a red mitochondria-specific cationic fluorescent dye (Mitotracer Deep Reed, Life Technologies, Foster, CA, USA) which accumulates in mitochondria depending on the ΔΨ, for 15 min at 37 °C. Once inside the mitochondria, the dye cannot flow back out irrespective of the mitochondrial membrane potential. The spectral characteristics of the harvested cells were analyzed by FACS using BD LSRFortessa™ (BD) Cytometer (excitation 644 nm, emission 665 nm).

### 4.14. Determination of 3T3-L1 Cellular ADP/ATP Ratio

3T3-L1 preadipocytes were differentiated and treated for 72 h. A luminometric ATP Assay kit (MAK135 Sigma-Aldrich, St. Louis, MO, USA) was used to measure ATP and ADP according to the manufacturers’ instructions. Briefly, we added 90 µL of ATP reagent to each well, incubated for 1 min at room temperature, and read luminescence using a Varioskan™ LUX multimode microplate reader (Thermo-Fisher Scientific, Waltham, MA, USA). We incubated the plate for an additional 10 min and read luminescence again. We added 5 µL of ADP reagent to each well and after 1 min, we read luminescence. We used the luminescence data to calculate ADP/ATP ratio.

### 4.15. Western Blot Analysis

Whole 3T3-L1 cell lysates were obtained by solubilizing cells in protein loading buffer and heating at 100 °C for 10 min. An equal amount of proteins from each sample was separated by sodium dodecyl sulfate–polyacrylamide gel electrophoresis, and the gels were transferred onto Hybond-P membranes (GE Healthcare Life Sciences, Piscataway, NJ, USA). Membranes were incubated with the antibodies for p-AMPK, total-AMPK, and Actin from Cell Signaling Technology (Danvers, MA, USA). Signal was detected by incubating the membranes with secondary antibodies labeled with horseradish peroxidase and revealed with the SuperSignal™ West Pico PLUS Chemiluminescent Substrate (Thermo- Fisher Scientific, Waltham, MA, USA).

### 4.16. Statistical Analysis

Data are presented as means ± SEM. Comparisons between groups were analyzed using one-way analysis of variance (ANOVA) followed by a Dunnett post hoc test. Statistical significance was set at *p* < 0.05. Analyses were performed with the Prism 4 (GraphPad software Inc.) statistical software.

## 5. Conclusions

UAL treatment reverted metabolic alterations in different cell types involved in the development of insulin resistance and T2DM. This triterpenoid had anti-inflammatory effects in macrophages and antilipogenic activity in hepatocytes and adipocytes, inhibiting adipogenic/lipogenic gene expression, overexpressing genes involved in the brown fat phenotype, and increasing AMPK phosphorylation. UAL presented potential hypoglycemic activity, with an increase in glucose uptake in insulin resistance myocytes simulating the conditions found in obese people. It is important to find new molecules that may improve biodisponibility and therapeutic effects. The multitarget biological activities shown here in vitro infer that UAL is a promising molecule for diabetes treatment and that further studies are needed to determine UAL effects in more complex models.

## Data Availability

Not applicable.

## Supplementary Materials

Table S1: Primer sequences for the targeted mouse genes.

## Sample Availability

Samples of UAL are available from the authors.

## References

[1] de Freitas Junior L.M., de Almeida E.B. (2017). Medicinal plants for the treatment of obesity: Ethnopharmacological approach and chemical and biological studies. Am. J. Transl. Res..

[2] International Diabetes Federation (2019). IDF Diabetes Atla.

[3] Schnurr T.M., Jakupović H., Carrasquilla G.D., Ängquist L., Grarup N., Sørensen T.I.A., Tjønneland A., Overvad K., Pedersen O., Hansen T. (2020). Obesity, unfavourable lifestyle and genetic risk of type 2 diabetes: A case-cohort study. Diabetologia.

[4] Smith U., Kahn B.B. (2016). Adipose tissue regulates insulin sensitivity: Role of adipogenesis, de novo lipogenesis and novel lipids. J. Intern. Med..

[5] Ríos J.L., Francini F., Schinella G.R. (2015). Natural products for the treatment of type 2 diabetes mellitus. Planta Med..

[6] Xu L., Li Y., Dai Y., Peng J. (2018). Natural products for the treatment of type 2 diabetes mellitus: Pharmacology and mechanisms. Pharmacol. Res..

[7] Alqahtani A., Hamid K., Kam A., Wong K.H., Abdelhak Z., Razmovski-Naumovski V., Chan K., Li K.M., Groundwater P.W., Li G.Q. (2013). The pentacyclic triterpenoids in herbal medicines and their pharmacological activities in diabetes and diabetic complications. Curr. Med. Chem..

[8] Furtado N.A.J.C., Pirson L., Edelberg H., Miranda L.M., Loira-Pastoriza C., Preat V., Larondelle Y., André C.M. (2017). Pentacyclic triterpene bioavailability: An overview of in vitro and in vivo studies. Molecules.

[9] Dey B., Mitra A. (2013). Chemo-profiling of eucalyptus and study of its hypoglycemic potential. World J. Diabetes.

[10] Kumar R., Rana S., Singh A. (2020). A review on phytochemistry and pharmacological profile of *Eucalyptus globulus*. Int. J. Pharm. Res..

[11] Guillén A., Granados S., Rivas K.E., Estrada O., Echeverri L.F., Balcázar N. (2015). Antihyperglycemic activity of eucalyptus tereticornis in insulin-resistant cells and a nutritional model of diabetic mice. Adv. Pharmacol. Sci..

[12] Ceballos S., Guilí En A., Lorena D., Noz M., Castã A., Echeverri L.F., Acín S., Bal N., Bal Azar N. (2018). Immunometabolic regulation by triterpenes of Eucalyptus tereticornis in adipose tissue cell line models. Phytomedicine.

[13] Acín S., Muñoz D.L., Guillen A., Soscue D., Castaño A., Echeverri F., Balcazar N. (2021). Triterpene-enriched fractions from Eucalyptus tereticornis ameliorate metabolic alterations in a mouse model of diet-induced obesity. J. Ethnopharmacol..

[14] Seo D.Y., Lee S.R., Heo J.W., No M.H., Rhee B.D., Ko K.S., Kwak H.B., Han J. (2018). Ursolic acid in health and disease. Korean J. Physiol. Pharmacol..

[15] Ramírez-Rodríguez A.M., González-Ortiz M., Martínez-Abundis E., Acuña Ortega N. (2017). Effect of ursolic acid on metabolic syndrome, insulin sensitivity, and inflammation. J. Med. Food.

[16] Castellano J.M., Guinda A., Delgado T., Rada M., Cayuela J.A. (2013). Biochemical basis of the antidiabetic activity of oleanolic acid and related pentacyclic triterpenes. Diabetes.

[17] Ayeleso T.B., Matumba M.G., Mukwevho E. (2017). Oleanolic acid and its derivatives: Biological activities and therapeutic potential in chronic diseases. Molecules.

[18] Maurya A., Srivastava S.K. (2012). Determination of ursolic acid and ursolic acid lactone in the leaves of eucalyptus tereticornis by hplc. J. Braz. Chem. Soc..

[19] Zatterale F., Longo M., Naderi J., Raciti G.A., Desiderio A., Miele C., Beguinot F. (2020). Chronic adipose tissue inflammation linking obesity to insulin resistance and type 2 diabetes. Front. Physiol..

[20] Ebbert J.O., Jensen M.D. (2013). Fat depots, free fatty acids, and dyslipidemia. Nutrients.

[21] Marica Bakovic N.H. (2015). Biologically active triterpenoids and their cardioprotective and anti- inflammatory effects. J. Bioanal. Biomed..

[22] Mlala S., Oyedeji A.O., Gondwe M., Oyedeji O.O. (2019). Ursolic acid and its derivatives as bioactive agents. Molecules.

[23] Bano Z., Begum S., Ali S.S., Kiran Z., Siddiqui B.S., Ahmed A., Khawaja S., Fatima F., Jabeen A. (2021). Phytochemicals from Carissa carandas with potent cytotoxic and anti-inflammatory activities. Nat. Prod. Res..

[24] He Y., Li Y., Zhao T., Wang Y., Sun C. (2013). Ursolic acid inhibits adipogenesis in 3T3-L1 adipocytes through LKB1/AMPK pathway. PLoS ONE.

[25] Zhu Y., Li X., Chen J., Chen T., Shi Z., Lei M., Zhang Y., Bai P., Li Y., Fei X. (2016). The pentacyclic triterpene Lupeol switches M1 macrophages to M2 and ameliorates experimental inflammatory bowel disease. Int. Immunopharmacol..

[26] Zhao J., Zheng H., Sui Z., Jing F., Quan X., Zhao W., Liu G. (2019). Ursolic acid exhibits anti-inflammatory effects through blocking TLR4-MyD88 pathway mediated by autophagy. Cytokine.

[27] Yadav V.R., Prasad S., Sung B., Kannappan R., Aggarwal B.B. (2010). Targeting inflammatory pathways by triterpenoids for prevention and treatment of cancer. Toxins.

[28] López-García S., Castañeda-Sanchez J.I., Jiménez-Arellanes A., Domínguez-López L., Castro-Mussot M.E., Hernández-Sanchéz J., Luna-Herrera J. (2015). Macrophage activation by ursolic and oleanolic acids during mycobacterial infection. Molecules.

[29] Podder B., Jang W.S., Nam K.W., Lee B.E., Song H.Y. (2015). Ursolic acid activates intracellular killing effect of macrophages during mycobacterium tuberculosis infection. J. Microbiol. Biotechnol..

[30] Starkov A.A. (2008). The role of mitochondria in reactive oxygen species metabolism and signaling. Proc. Ann. N. Y. Acad. Sci..

[31] Gong S., Peng Y., Jiang P., Wang M., Fan M., Wang X., Zhou H., Li H., Yan Q., Huang T. (2014). A deafness-associated tRNAHis mutation alters the mitochondrial function, ROS production and membrane potential. Nucleic Acids Res..

[32] Schieber M., Chandel N.S. (2014). ROS function in redox signaling and oxidative stress. Curr. Biol..

[33] Rendra E., Riabov V., Mossel D.M., Sevastyanova T., Harmsen M.C., Kzhyshkowska J. (2019). Reactive oxygen species (ROS) in macrophage activation and function in diabetes. Immunobiology.

[34] Lee M.S., Thuong P.T. (2010). Stimulation of glucose uptake by triterpenoids from Weigela subsessilis. Phyther. Res..

[35] Zhang W., Hong D., Zhou Y., Zhang Y., Shen Q., Li J.Y., Li J.-Y., Hu L.-H., Li J. (2006). Ursolic acid and its derivative inhibit protein tyrosine phosphatase 1B, enhancing insulin receptor phosphorylation and stimulating glucose uptake. Biochim. Biophys. Acta Gen. Subj..

[36] Chiang D.J., Pritchard M.T., Nagy L.E. (2011). Obesity, diabetes mellitus, and liver fibrosis. Am. J. Physiol. Gastrointest. Liver Physiol..

[37] Sharma H., Kumar P., Deshmukh R.R., Bishayee A., Kumar S. (2018). Pentacyclic triterpenes: New tools to fight metabolic syndrome. Phytomedicine.

[38] Zorova L.D., Popkov V.A., Plotnikov E.Y., Silachev D.N., Pevzner I.B., Jankauskas S.S., Babenko V.A., Zorov S.D., Balakireva A.V., Juhaszova M. (2018). Mitochondrial membrane potential. Anal. Biochem..

[39] Garcia D., Shaw R.J. (2017). AMPK: Mechanisms of cellular energy sensing and restoration of metabolic balance. Mol. Cell.

[40] Chen C., Deng Y., Hu X., Ren H., Zhu J., Fu S., Xie J., Peng Y. (2018). miR-128-3p regulates 3T3-L1 adipogenesis and lipolysis by targeting Pparg and Sertad2. J. Physiol. Biochem..

[41] Kim G.C., Kim J.S., Kim G.M., Choi S.Y. (2017). Anti-adipogenic effects of Tropaeolum majus (nasturtium) ethanol extract on 3T3-L1 cells. Food Nutr. Res..

[42] Fu Y., Luo N., Klein R.L., Garvey W.T. (2005). Adiponectin promotes adipocyte differentiation, insulin sensitivity, and lipid accumulation. J. Lipid Res..

[43] Barberá M.J., Schlüter A., Pedraza N., Iglesias R., Villarroya F., Giralt M. (2001). Peroxisome proliferator-activated receptor α activates transcription of the brown fat uncoupling protein-1 gene. A link between regulation of the thermogenic and lipid oxidation pathways in the brown fat cell. J. Biol. Chem..

[44] Corrales P., Vidal-Puig A., Medina-Gómez G. (2018). PPARS and metabolic disorders associated with challenged adipose tissue plasticity. Int. J. Mol. Sci..

[45] Pérez-Pérez A., Sánchez-Jiménez F., Vilariño-García T., Sánchez-Margalet V. (2020). Role of leptin in inflammation and vice versa. Int. J. Mol. Sci..

