# Supplementary material for: Ursolic Acid Lactone Obtained from Eucalyptus tereticornis Increases Glucose Uptake and Reduces Inflammatory Activity and Intracellular Neutral Fat: An In Vitro Study
Source: Molecules. 2021 Apr 15;26(8):2282. doi: 10.3390/molecules26082282 (PMC8071196; doi:10.3390/molecules26082282)
Supplement: Supplementary file 1 [file molecules-26-02282-s001.zip › molecules-1175335-supplementary.pdf]

Supplementary Material

Ursolic acid lactone obtained from Eucalyptus tereticornis increases glucose uptake and reduces inflammatory activity and intracellular neutral fat: an in vitro study

Norman Balcazar [1,3], Laura I. Betancur [1], Diana L. Muñoz[3], Frankly J. Cabrera[1], Adriana Castaño[2], Luis F. Echeverri[2] and Sergio Acin [1,3]*

[1] GENMOL Group. Sede de Investigación Universitaria, Cl. 62 #52-59, University of Antioquia 050010 Medellín, Antioquia, Colombia
[2] QOPN Group - Sede de Investigación Universitaria, Faculty of Exact and Natural Sciences, Cl. 62 #52-59, University of Antioquia, 050010 Medellín, Antioquia, Colombia
[3] Department of Physiology and Biochemistry, Faculty of Medicine, Cra. 51d #62-29, University of Antioquia, 050010 Medellín, Antioquia, Colombia
* Correspondence: sergio.acin@udea.edu.co

Table SI. Primer sequences for the targeted mouse genes

Gene	Name		Sequences	A.T.
PPARG	peroxisome proliferator activated receptor gamma	F	5´-CACTCGCATTCCTTTGAC-3´	53ºC
		R	5´-ACTTTGATCGCACTTTGG-3´	
ADIPOQ	Adiponectin	F	5´-CCCAAGGGAACTTGTGCAGGTTGGATG-3´	64ºC
		R	5´-GTTGGTATCATGGTAGAGAAGAAAGCC-3´	
LEP	Leptin	F	5´-GCCAGGCTGCCAGAATTG-3´	58ºC
		R	5´-CTGCCCCCCAGTTTGATG-3´	
ACACA	Acetyl-CoA carboxylase	F	5´- GCCTCTTCCTGACAAACGAG-3´	60ºC
		R	5´- TGACTGCCGAAACATCTCTG-3´	
UCP1	Uncoupling protein 1	F	5'-GGCATTCAGAGGCAAATCAGCT -3'	60ºC
		R	5'-CAATGAACACTGCCACACCTC -3'	
PPARA	Peroxisome proliferator activated receptor alpha	F	5´- TGTCGAATATGTGGGGACAA-3´	60ºC
		R	5´- AATCTTGCAGCTCCGATCAC-3´	
SREBF1	Sterol regulatory element binding protein 1	F	5´- ATCGGCGCGGAAGCTGTCGGGGTAG-3´	68ºC
		R	5´- ACTGTCTTGGTTGTTGATGAGCTGGA-3´	

FASN	Fatty Acid Synthase	F	5´- CCCTTGATGAAGAGGGATCA-3´	60ºC
		R	5´- ACTCCACAGGTGGGAACAAG-3´	
PPARGC 1A	PPARG Coactivator 1 Alpha	F	5´- TATGGAGTGACATAGAGTGTGCT-3´	60ºC
		R	5´- CCACTTCAATCCACCCAGAAAG-3´	
CEBPA	CCAAT Enhancer Binding Protein Alpha	F	5’- TGGACAAGAACAGCAACGAG-3’	59ºC
		R	5’- CCATGGCCTTGACCAAGGAG-3’	
TNF-α	Tumor Necrosis Factor	F	5’- CCCACACCGTCAGCCGATTT-3’	60ºC
		R	5’- GTCTAAGTACTTGGGCAGATTGACC-3’	
IL-1β	Interleukin 1 Beta	F	5’- AGAGCTTCAGGCAGGCAGTAT-3’	60ºC
		R	5’- GAAGGTGCTCATGTCCTCATC-3’	
IL-6	Interleukin 6	F	5’- CCGGAGAGGAGACTTCACAG-3’	60ºC
		R	5’- GGAAATTGGGGTAGGAAGGA-3’	
PPIB	Peptidylprolyl isomerase B	F	5´-GGAGATGGCACAGGAGGAA -3´	60ºC
	Cyclophilin B	R	5´-GTAGTGCTTCAGCTTGAAGTTCTCAT -3´	

Forward (F); Reverse (R); Anealing Temperature (A.T.)
